# Dynamic *In Vitro* Models of the Human Gastrointestinal Tract as Relevant Tools to Assess the Survival of Probiotic Strains and Their Interactions with Gut Microbiota

**DOI:** 10.3390/microorganisms3040725

**Published:** 2015-10-23

**Authors:** Charlotte Cordonnier, Jonathan Thévenot, Lucie Etienne-Mesmin, Sylvain Denis, Monique Alric, Valérie Livrelli, Stéphanie Blanquet-Diot

**Affiliations:** 1 Équipe d’Accueil Conception, Ingénierie et Développement de l’Aliment et du Médicament (EA 4678 CIDAM), Centre de Recherche en Nutrition Humaine Auvergne, Université d’Auvergne, Centre Biomédical de Recherche et de Valorisation (CBRV) 28 place Henri Dunant, 63000 Clermont-Ferrand, France; E-Mails: charlotte.cordonnier@udamail.fr (C.C.); thevenot.jonathan@gmail.com (J.T.); lucie.etiennemesmin@yahoo.fr (L.E.-M.); sylvain.denis@udamail.fr (S.D.); monique.alric@udamail.fr (M.A.); 2 Microbes, Intestin, Inflammation et Susceptibilité de l’Hôte (M2iSH), Unité Mixte de Recherche Institut National de la Santé Et de la Recherche Médicale (UMR INSERM) / Université d’Auvergne U1071 Unité Sous Contrat - Institut National de Recherche Agronomique (USC-INRA) 2018, Centre de Recherche en Nutrition Humaine Auvergne, Université d’Auvergne, CBRV 28 place Henri Dunant, 63000 Clermont-Ferrand, France; E-Mail: valerie.livrelli@udamail.fr; 3 Service de Bactériologie, Centre Hospitalier Universitaire (CHU), Clermont-Ferrand, 58 rue Montalembert, 63000 Clermont-Ferrand, France

**Keywords:** probiotic, *Saccharomyces cerevisiae*, survival, *in vitro* models, human gastrointestinal tract, intestinal microbiota, food matrix

## Abstract

The beneficial effects of probiotics are conditioned by their survival during passage through the human gastrointestinal tract and their ability to favorably influence gut microbiota. The main objective of this study was to use dynamic *in vitro* models of the human digestive tract to investigate the effect of fasted or fed state on the survival kinetics of the new probiotic *Saccharomyces cerevisiae* strain CNCM I-3856 and to assess its influence on intestinal microbiota composition and activity. The probiotic yeast showed a high survival rate in the upper gastrointestinal tract whatever the route of admistration, *i.e.*, within a glass of water or a Western-type meal. *S. cerevisiae* CNCM I-3856 was more sensitive to colonic conditions, as the strain was not able to colonize within the bioreactor despite a twice daily administration. The main bacterial populations of the gut microbiota, as well as the production of short chain fatty acids were not influenced by the probiotic treatment. However, the effect of the probiotic on the gut microbiota was found to be individual dependent. This study shows that dynamic *in vitro* models can be advantageously used to provide useful insight into the behavior of probiotic strains in the human digestive environment.

## 1. Introduction

Probiotics are defined as “live microorganisms that, when administered in adequate amounts, confer a health benefit on the host” [1]. The most commonly used probiotics are lactic acid bacteria such as *Lactobacilli*, *Enterococci* or *Bifidobacteria*. Even if most studies about probiotics have focused primarily on bacteria, there are also many reports showing the potential of probiotic yeasts [2]. Among them, *Saccharomyces cerevisiae* var *boulardii* has long been known to be effective for treating acute and chronic intestinal diseases [3,4,5]. The main mechanisms of action of probiotic yeast are (i) the direct or indirect inhibition of intestinal pathogens, (ii) the modification of host signaling pathways, especially those involved in inflammatory response, (iii) the stimulation of the immune system, and (iv) the trophic effects on intestinal mucosa [3,4,5].

Although only *S. boulardii* has been widely studied and its inhibitory mechanisms are well defined, other yeast strains have been considered for their probiotic properties. In particular, *Saccharomyces cerevisiae* CNCM I-3856 is a new probiotic yeast, which has been shown to decrease inflammation in a mouse model of chemically-induced colitis [6], to prevent colitis induced by AIEC (adherent-invasive *Escherichia coli*) in the transgenic mice model mimicking Crohn’s disease [7] and to reduce digestive discomfort and abdominal pain in patients with irritable bowel syndrome [8]. *Saccharomyces cerevisiae* CNCM I-3856 has also shown interesting antagonistic properties against other pathogenic *Escherichia coli*, such as ETEC (enterotoxigenic *E. coli*) and EHEC (enterohemorrhagic *E. coli*). Zanello *et al.*, have shown that viable *S. cerevisiae* CNCM I-3856 inhibits the ETEC-induced pro-inflammatory pathways in porcine intestinal epithelial cells [9]. Using relevant dynamic *in vitro* models of the upper and lower human gastrointestinal tract, Etienne-Mesmin *et al.*, and Thévenot *et al.*, have revealed that this probiotic yeast significantly reduces the growth resumption of EHEC O157:H7 observed in the distal parts of the small intestine and decreases Shiga-toxin expression in the large intestine [10,11,12].

Although the above studies have provided evidence of the effectiveness of *S. cerevisiae* CNCM I-3856, the mode of action of this probiotic yeast still needs to be clarified. In particular, very little published data are available on its survival in the human digestive environment [10,13], and information about its interactions with human microbiota is clearly missing, although these two parameters are key features of probiotic strains.

In this context, the aim of the present study was to use the potential of dynamic *in vitro* digestion models to expand the current knowledge on the behavior of *S. cerevisiae* CNCM I-3856 in the human digestive tract. In the first step, the influence of fed or fasted conditions on *Saccharomyces cerevisiae* CNCM I-3856 survival in the upper human gastrointestinal tract was investigated using the gastric and small intestinal model TIM-1 (TNO gastroIntestinal Model-1). The probiotic showed a high resistance to the gastric and small intestine environment whatever the mode of administration. In a second step, the yeast survival in human colonic conditions was assessed using the ARCOL (artificial colon) model, as well as its effects on human gut microbiota composition and metabolic activity. *S. cerevisiae* CNCM I-3856 was not able to colonize in the large intestinal conditions but had an individual-dependent effect on gut microbiota profiles. TIM-1 and ARCOL provide clarification on the behavior of the probiotic yeast strain during digestion in humans.

## 2. Experimental Section

### 2.1. Yeast Strain

The yeast strain *S. cerevisiae* CNCM I-3856 (Lynside Pro GI${}^{+}$, Lesaffre Human Care, Marcq-en-Baroeul, France) was supplied in its active dried powder form and administered into the TIM-1 and ARCOL at a concentration of 10${}^{7}$ CFU/mL.

### 2.2. Simulated Human Digestive Conditions

#### 2.2.1. *In Vitro* Digestion in the TIM-1 System

The gastro-intestinal TIM-1 system (TNO, Zeist, The Netherlands) is a multi-compartmental, dynamic, computer-controlled model that simulates the upper human gastro-intestinal tract (Table 1). TIM-1 consists of four successive compartments simulating the conditions found in the stomach and the three segments of the small intestine in humans, *i.e.*, the duodenum, jejunum, and ileum. The main parameters of human digestion, such as pH, body temperature, peristaltic mixing and transport, gastric, biliary and pancreatic secretions, and passive absorption of small molecules and water, are reproduced as accurately as possible. Briefly, each compartment is composed of glass units with a flexible inner membrane. Peristaltic mixing and body temperature are achieved by pumping water at 37 °C into the space between the glass jacket and the flexible wall at regular intervals. Mathematical modeling of gastric and ileal deliveries with a power exponential equation [14] is used for the computer control of chyme transit. In the Elashoff equation ($f=1-2^{-{\left(\frac{t}{t_{1/2}}\right)}^{\beta }}$), $t_{1/2}$ is the half time of gastric or ileal emptying and *β* a coefficient describing the shape of the curve. Chyme transport through the TIM-1 is regulated by the peristaltic valves that connect the successive compartments. The volume in each compartment is monitored by a pressure sensor, and pH is computer-monitored and continuously controlled by adding either HCl (gastric compartment) or NaHCO_3_ (intestinal compartments). Simulated gastric, biliary and pancreatic secretions are introduced into the corresponding compartments by computer-controlled pumps. Water and products of digestion are removed from the jejunal and ileal compartments by pumping dialysis liquid through hollow fiber membranes (SF 90G, Nipro^®^, Osaka, Japan, with a molecular mass cut-off value of 10 kDa). Before each experiment, the system is washed with detergent, rinsed with water and decontaminated by steaming at 105 °C for 45 min.

**Table 1:** Schematic representation of TIM-1 and ARCOL.

*In Vitro* Models		Main Parameters
**TIM-1** (gastric and smallintestinal model)	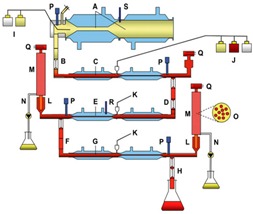	- body temperature - evolution of gastric and intestinal pH - chyme mixing - transit time - gastric and ileal deliveries - gastric, biliary and pancreatic secretions - passive absorption of water and digestion products
**ARCOL** (large intestinal model)	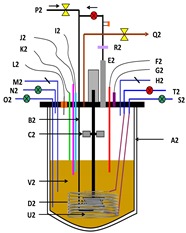	- body temperature - colonic pH - large intestine retention time - supply of ileal effluents - anaerobiosis maintained by resident microbiota activity - passive absorption of water and fermentation metabolites

Schematic representation of TIM-1 from [15]: A: gastric compartment; B: pyloric sphincter; C: duodenal compartment; D: peristaltic valves; E: jejunal compartment; F: peristaltic valves; G: ileal compartment; H: ileal-cecal valve; I: gastric secretion (lipase, pepsin); J: duodenal secretion (bile, pancreatic juice, electrolytes); K: bicarbonate secretion; L: pre-filter; M: filtration system; N: filtrate with bio-accessible fraction; O: hollow fiber system (cross section); P: pH electrodes; Q: level sensors; R: temperature sensors; S: pressure sensor. Schematic representation of ARCOL: A2: reactor with double thermal jacket; B2: sparger; C2: rushton impeller; D2: marine impeller; E2: condenser; F2: redox sensor; G2: pressure control; H2: inoculum inlet; I2: pH sensor; J2: temperature sensor; K2: level sensor; L2: NaOH inlet; M2: sampling system; N2: medium inlet; O2: medium outlet; P2: N_2_ (only before inoculation with fecal sample); Q2: gas outlet; R2: filter system; S2: dialysis inlet; T2: dialysis outlet; U2: dialysis fiber; V2: colonic medium.

The TIM-1 system was programmed to reproduce according to *in vivo* data the physicochemical conditions observed during the digestion of a glass of water (fasted state, *n* = 3) or a solid meal (fed state, *n* = 4) in a healthy human adult (Table 2). The total duration of the experiments was 300 min. In the fasted state, the suspension (200 mL) that was introduced into the TIM-1 system consisted of mineral water (Volvic^®^, Danone, Volvic, France) inoculated with *S. cerevisiae* CNCM I-3856 in powder form (final concentration 10${}^{7}$ CFU/mL). The test meal used in the fed protocol was composed of 16.3 g of mixed diced vegetables, 2.5 g of salad dressing, 25 g of undercooked ground beef, 7.8 g of instant mashed potato, 16.3 g of Ultra High Temperature (UHT) full-cream milk, 4 g of cream cheese, 25 g of applesauce and 15 g of sliced white bread. The volume of the meal was adjusted to 300 mL with mineral water (Volvic^®^, France), homogenized for 20 min with an Ultra Turrax system (T25, IKA^®^, Werke, Staufen, Germany) set at 24,000 rpm before inoculation of *S. cerevisiae* CNCM I-3856 in powder form (final concentration 10${}^{7}$ CFU/mL). Samples were taken in the initial suspensions (glass of water or solid meal) before introduction into the artificial stomach and regularly collected during digestion in the different compartments of the TIM-1 system, as well as in the cumulative ileal effluents kept on ice and pooled hour-by-hour in order to determine the survival rate of the probiotic yeast in the upper gastrointestinal tract. Control digestions with a transit marker were carried out under the same experimental conditions (*n* = 3 with fasted protocol, *n* = 4 with fed protocol) by using water containing 0.8% (wt/vol) of blue dextran as the initial suspension [16].

#### 2.2.2. *In Vitro* Fermentation in the ARCOL Model

ARCOL is a one-stage semi-continuous fermentation system (Applikon, Schiedam, The Netherlands) that integrates the main parameters of the *in vivo* human colonic environment, including pH, body temperature, supply of ileal effluents, retention time, anaerobiosis maintained by the sole activity of resident microbiota, and passive absorption of water and fermentation metabolites [12] (Table 1). Fresh feces from healthy volunteers who had no history of antibiotic or probiotic treatment 3 months before the study were used to inoculate the bioreactor. The fecal inoculum was prepared under strict anaerobic conditions in a vinyl anaerobic chamber (Coy, Grass Lake, MI, USA). Stools (≈ 50 g) were mixed with 350 mL of a 200 mM sodium phosphate buffer and filtered through a double layer of gauze. The fecal suspension was rapidly transferred to the bioreactor, flushed with $O_{2}$-free $N_{2}$ gas, and brought to 450 mL with culture medium. The ARCOL model was run under conditions reproducing a healthy human colon with a fixed temperature of 37 °C, a controlled constant pH of 6.3, a mean retention time of 36 h and a redox potential (*Eh*) of –400 mV. The nutritive medium, which was sequentially introduced into the bioreactor, contained various carbohydrate, protein, lipid, mineral and vitamin sources, in order to closely mimic the composition of the ileal effluents [17]. The fermentative process allowed the maintenance of anaerobic conditions in the bioreactor, with the initial sparging with $O_{2}$-free $N_{2}$ gas being stopped after inoculation. A dialysis system using hollow fiber membranes (molecular mass cut-off value of 30 kDa) maintained the appropriate electrolyte and metabolite concentrations and the operating volume. Two experimental schemes (*n* = 3 for each condition) were used: (i) no supplementation and (ii) twice daily supplementation with *S. cerevisiae* CNCM I-3856 re-suspended in sterile saline water (final concentration in the bioreactor of 10${}^{7}$ CFU/mL). Each experiment started after a 4-day stabilization phase and was done in triplicate using feces collected from three different volunteers. Samples were taken immediately after inoculation of the probiotic yeast and regularly collected from the colonic medium to determine its survival kinetics in the large intestine. In parallel, the main bacterial populations of human intestinal microbiota were followed by real-time quantitative PCR (qPCR) analysis. Dialysis outflows of the ARCOL model were daily sampled to determine short chain fatty acid (SCFA) production by gas chromatography.

**Table 2:** Parameters of *in vitro* digestions in the TIM-1 model.

*In Vitro* Digestion Parameters	Fasted – “Glass of Water” Protocol	Fed – “Solid Meal” Protocol
pH	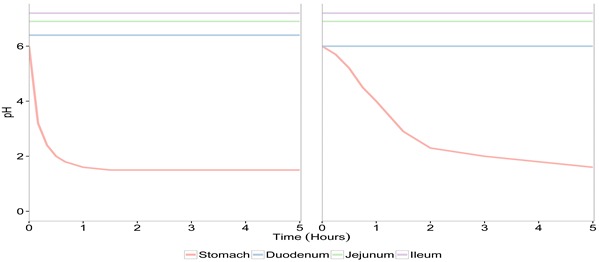	
Transit time	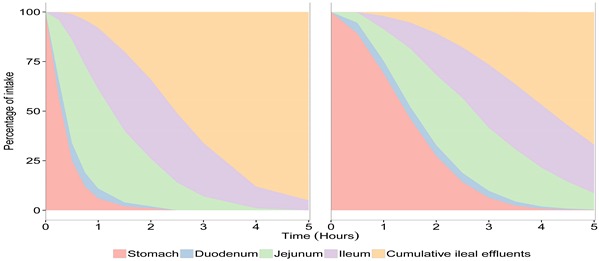	
	Gastric compartment	
	130 U/min of pepsin	520 U/min of pepsin
	5 U/min of lipase	20 U/min of lipase
	0.25 mL/min of HCl 0.3 M if necessary	0.25 mL/min of HCl 1.5 M if necessary
	β=1 and $t_{1/2}=15$ min	β=1.8 and $t_{1/2}=85$ min
Digestive secretions	Duodenal compartment	
	20 mg/min of bile extract during 30 min of digestion then 10 mg/min	
	0.25 mg/min of intestinal electrolyte solution	
	20 mg/min of pancreatin (4 USP)	80 mg/min of pancreatin (4 USP)
	0.25 mL/min of NaHCO_3_ 0.5 M if necessary	0.25 mL/min of NaHCO_3_ 1 M if necessary
	Jejunal compartment	
	0.25 mL/min of NaHCO_3_ 0.5 M if necessary	0.25 mL/min of NaHCO_3_ 1 M if necessary
	Ileal compartment	
	0.25 mL/min of NaHCO_3_ 0.5 M if necessary	0.25 mL/min of NaHCO_3_ 1 M if necessary
	β=2.4 and $t_{1/2}=150$ min	β=2.5 and $t_{1/2}=250$ min
Dialysis (Jejunum and ileum)	10 mL/min	

U: unity; USP: United States Pharmacopeia. The Elashoff equation ($f=1-2^{-{\left(\frac{t}{t_{1/2}}\right)}^{\beta }}$), where *f* represents the fraction of meal delivered and *t* the time of delivery, *t*_1/2_ the half-time of delivery, and *β* a coefficient describing the shape of the curve, was used for the computer control of gastric and ileal deliveries in the TIM-1 system [14].

### 2.3. Yeast Survival in the *in Vitro* Gastrointestinal Tract

The survival of *S. cerevisiae* CNCM I-3856 in the TIM-1 and ARCOL systems was determined by plating on Sabouraud dextrose agar supplemented with gentamicin (10 μg/L) and chloramphenicol (50 μg/L) followed by incubation at 30 °C during 48 h. The number of culturable cells was determined by visual counting and used as an indicator of probiotic survival. In the TIM-1 system, results were expressed as percentages of initial intake and were cross-compared to those obtained with the blue dextran transit marker. This compound is a non-absorbable transit marker, which indicates a 100% survival percentage for yeast. Its concentrations throughout the TIM-1 fluctuate according to the volume of each digestive compartment, the rate of dilution by digestive secretions and the chyme flow between two successive compartments. Thereby, yeast curves below that of the transit marker will reflect cell mortality, while curves above the transit marker will be indicative of yeast growth renewal. Concentrations of blue dextran in the digestive samples from the TIM-1 system were determined colorimetrically using a Multisckan spectrum (Thermo Scientific, Schaumburg, IL, USA) at 595 nm. In the ARCOL system, results were expressed as log${}_{10}$ colony forming units (CFU)/mL. Bacterial concentrations were normalized with respect to the amount on the last day of the stabilization phase.

### 2.4. Composition and Metabolic Activity of Human Colonic Microbiota

Total genomic DNA was extracted from 250 μL of colonic medium by using the two first steps of Yu and Morrison’s protocol [18] and the QIAamp DNA stool minikit (Qiagen, Courtaboeuf, France), according to the manufacturer’s instructions. The main bacterial populations of human intestinal microbiota (*Bacteroidetes*, *Firmicutes*, *Actinobacteria*, *Bifidobacteria*, *Bacteroides*, *Enterobacteriaceae*, *Lactococcus*/*Pediococcus*/*Leuconostoc*, *E. coli*, *Faecalibacterium prausnitzii*) were followed in the ARCOL model by qPCR analysis, as previously described by Thévenot *et al.* [12]. Primers used in this study are listed in Table 3.

SCFAs production in the ARCOL model was determinated by gas chromatography. Dialysis outflows supplemented with internal standard (2-ethyl butyric acid at 49 mmol/L) were deproteinized by the addition of saturated phosphotungstic acid (500 g/L), centrifuged at 9000 × *g* for 20 min before supernatants were filtered (0.45 μg/L). The samples were run through a Agilent 6890 Series GC System (Agilent Technologies, Massy, France) fitted with an HP-INNOWax column (0.25 mm × 30 m × 0.25 μm) and a flame ionization detector. Helium was used as a carrier gas at a flow rate of 2 mL/min. The injector and detector were set at 250 °C. The column was maintained in an oven with a temperature gradient ranging from 110 to 240 °C. One microliter quantity of each sample was injected with a run time of 14.3 min. Peaks were integrated using the HP ChemStation software. SCFA concentrations were quantified by comparing their peak areas with the corresponding standards.

**Table 3:** Primer and probe sequences used in real-time qPCR assays.

Name	Sequence 5’–3’	Target	Annealing	References
			Temperature (°C)	
SYBR green				
BAC338F	ACTCCTACGGGAGGCAG	Total bacteria	58	[19]
BAC338F	GTATTACCGCGGCTGCTG			
789cfbF	CRAACAGGATTAGATACCCT	*Bacteroidetes*	61	[20]
cfb967R	GGTAAGGTTCCTCGCGTAT			
Act920F3	TACGGCCGCAAGGCTA	*Actinobacteria*	61	[20]
Act1200R	TCRTCCCCACCTTCCTCCG			
928F-Firm	TGAAACTYAAAGGAATTGACG	*Firmicutes*	61	[20]
1040FirmR	ACCATGCACCACCTGTC			
Eco1457F	CATTGACGTTACCCGCAGAAGAAGC	*Enterobacteriaceae*	63	[21]
Eco1652R	CTCTACGAGACTCAAGCTTGC			
F_Lacto05	AGCAGTAGGGAATCTTCCA	*Lactobacillus/Pediococcus/Leuconostoc*	60	[22]
R_Lacto04	CGCCACTGGTGTCTYTCCATATA			
F_Fpra 428	TGTAAACTCCTGTTGTTGAGGAAGATAA	*Faecalibacterium prausnitzii*	60	[23]
R_Fpra 583	GCGCTCCCTTTACACCCA			
TaqMan				
F_Bact 1369	CGGTGAATACGTTCCCGG	Total bacteria	60	[22]
P_TM1389F	FAM-CTTGTACACACCGCCCGTC-TAMRA			
R_Prok1492R	TACGGCTACCTTGTTACGACTT			
E. coli-F	CATGCCGCGTGTATGAAGAA	*Escherichia coli*	60	[24]
E. coli-P	FAM-TATTAACTTTACTCCCTTCCTCCCCGCTGAA-TAMRA			
E. coli-R	CGGGTAACGTCAATGAGCAAA			
F_Bifid 09c	CGGGTGAGTAATGCGTGACC	Bifidobacteria	60	[22]
P_Bifid	FAM-CTCCTGGAAACGGGTG-TAMRA			
R_Bifid 06	TGATAGGACGCGACCCCA			
F_Bacter 11	CCTWCGATGGATAGGGGTT	*Bacteroides/Prevotella*	60	[22]
P_Bac303	YY-AAGGTCCCCCACATTG-TAMRA			
R_Bacter 08	CACGCTACTTGGCTGGTTCAG			

### 2.5. Statistical Analysis

For all experiments, significant differences in survival between treatments and time points were tested using a nonparametric analysis of repeated measures with the “f1.ld.f1” function of the package “nparLD” [25] in R 3.1.2 [26]. In case of a significant treatment effect, the function “npar.t.test” of the package “nparcomp” [27] was used for each time point. In case of a significant interaction effect, a linear mixed effect model with a random intercept on experiments to take into account the repeated measures was performed and followed by function “difflsmeans” of the package “lmerTest” [28]. The agglomerative hierarchical clustering with the “hclust” function was used to cluster treatments depending on their composition in major bacterial populations (*Bacteroidetes*, *Firmicutes*, *Actinobacteria*, *Bacteroides*, *Lactococcus*/*Pediococcus*/*Leuconostoc*, *Enterobacteriaceae*).

## 3. Results

### 3.1. Yeast Viability in the Upper Gastro-Intestinal Tract

**Figure 1:**
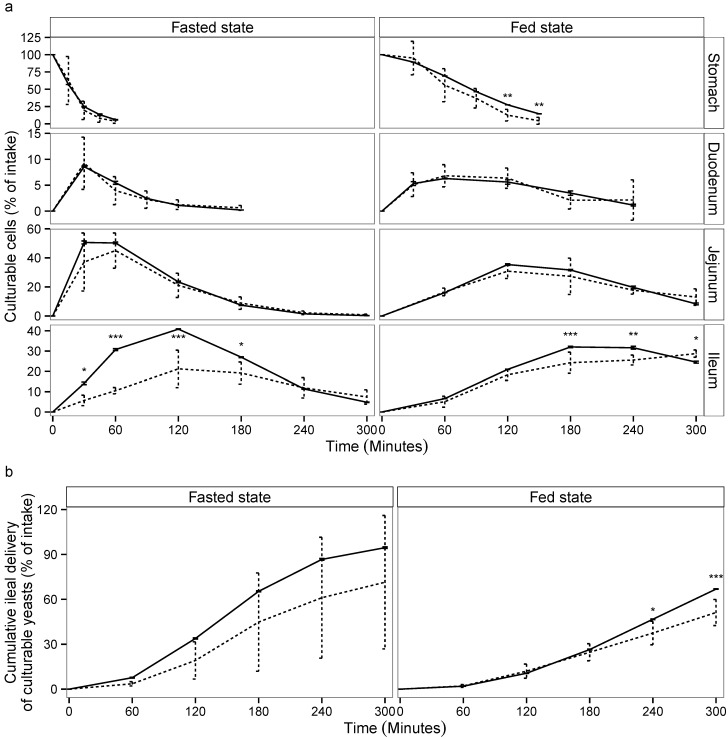
Survival of *S. cerevisiae* CNCM I-3856 in the gastro-intestinal TIM-1 system when ingested within a glass of water (“fasted state”) or a Western diet meal (“fed state”). Recovery profiles of yeast (dotted line) in the digestive compartments (**a**) and in the cumulative ileal effluents (**b**) are compared to that of the blue dextran transit marker (solid line). Values are given as mean percentages of initial intake ± the standard deviations (*n* = 3 for the fasted protocol, *n* = 4 for the fed protocol). Significant differences between yeast and transit marker are indicated (*p* < 0.05 (⋆), *p* < 0.01 (⋆⋆), *p* < 0.001 (⋆⋆⋆)).

The viability of *S. cerevisiae* CNCM I-3856 was evaluated in each compartment of TIM-1 (Figure 1a) and in the ileal effluents (Figure 1b) by cross-comparing the curves obtained for the yeast and the blue dextran transit marker in the two experimental conditions (fasted and fed state). When the probiotic was administered within a glass of water, the yeasts showed a high resistance to gastric, duodenal and jejunal conditions as no significant differences could be noticed between the curves obtained for the probiotic yeast and the transit marker (*p* > 0.05). In the ileal compartment, cell mortality was observed from 30 to 180 min of digestion (*p* < 0.05). When the probiotic was administered with the solid meal, it was found to be sensitive to the gastric conditions, since cell mortality was observed from 120 min digestion (*p* < 0.01). In the small intestinal compartments, yeast recovery followed a similar trend to that of the transit marker, except for the ileal compartment where the yeast population significantly decreased at 180 and 240 min of digestion (*p* < 0.05). From 180 min of digestion, the probiotic was recovered longer in the jejunum and ileum under the fed state, due to slower transit time.

The cumulative ileal delivery of culturable yeasts is shown in Figure 1b. Under the fasted condition, no significant differences were observed between the yeast and the transit marker. At the end of digestion (300 min), 71.4% ± 44.6% of the initial amount of yeast was recovered in the ileal effluents compared to 95.5 %± 0.5% for the transit marker (*p* > 0.05). Under the fed condition, a significant loss of viability was observed for *S. cerevisiae* CNCM I-3856 from 240 min of digestion (*p* < 0.05). At the end of digestion, 51.2% ± 8.7% of the initial amount of yeast was recovered in the ileal effluents compared to 66.9% ± 0.1% for the transit marker (*p* < 0.001). Under the fasted and fed state, the amount of culturable yeasts likely to reach the large intestine was 6.1 ± 0.01 and 6.8 ± 0.09 log${}_{10}$ CFU/mL, respectively.

**Figure 2:**
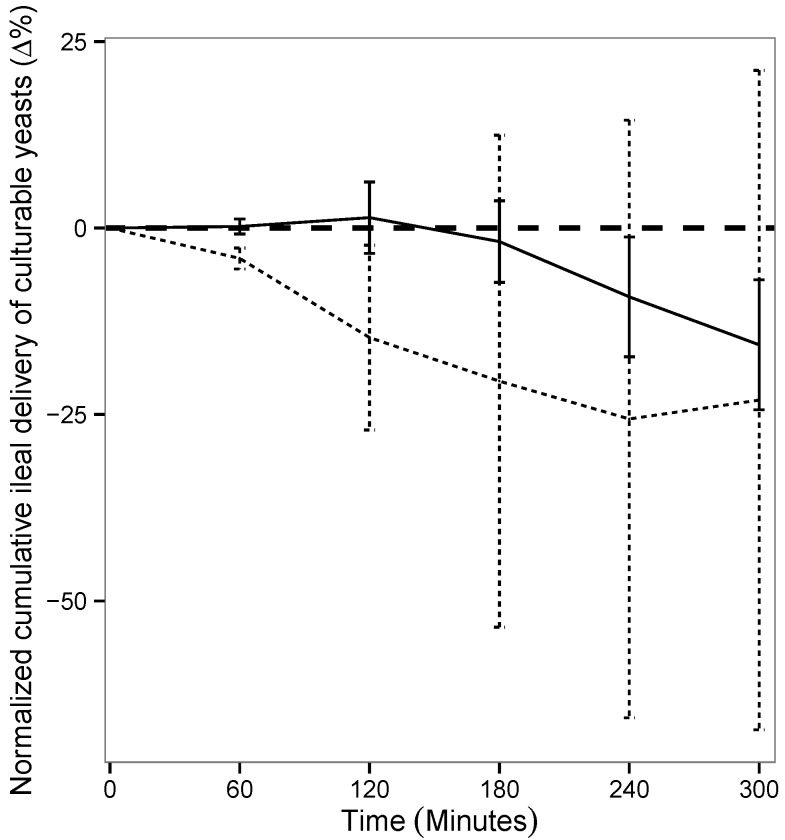
Influence of food matrix on yeast survival rate in the ileal effluents of the TIM-1 system. Recovery profiles of yeast under fasted (dotted line) and fed conditions (solid line) are indicated. Values are given as mean percentages of viable yeast normalized to the transit marker at each time point (∆%) ± the standard deviations (*n* = 3 for the fasted protocol, *n* = 4 for the fed protocol).

As in the TIM-1 system the transit flow, and consequently, the recovery percentages of the transit marker depend on the digestive protocol (fed or fasted), the results obtained for the probiotic yeast were normalized to the values of blue dextran in each condition. This allowed us to really assess the impact of food matrix on the survival of *S. cerevisiae* CNCM I-3856 in the *in vitro* human gastrointestinal tract. Figure 2 shows that yeast survival rate in the ileal effluents of the TIM-1 system was not influenced by the routes of administration, *i.e.*, within a glass of water or a complete meal (*p* > 0.05).

### 3.2. Yeast Viability in the Lower Gastro-Intestinal Tract

**Figure 3:**
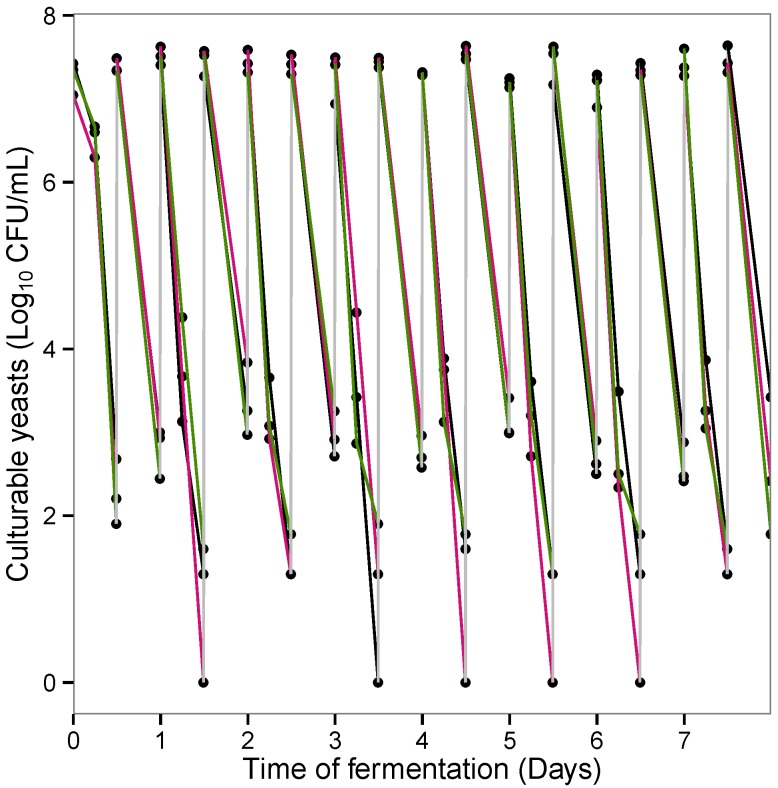
Survival kinetics of *S. cerevisiae* CNCM I-3856 in the ARCOL model after twice daily administration of the probiotic. Results are expressed in log${}_{10}$ CFU/mL for Donors 1 (in black), 2 (in pink) and 3 (in green).

Figure 3 shows the number of culturable *S. cerevisiae* CNCM I-3856 in the ARCOL model following twice daily pulse administration of the probiotic. Following each injection, yeast concentrations immediately reached a peak level around 7log ${}_{10}$ CFU/mL. However, these initial levels did not persist in the colonic medium. Indeed, the probiotic was quickly cleared from the bioreactor after each injection (except for the first one) and most times disappeared from the colonic medium between two consecutive additions. On average, at 12 h post administration, from 0 to 2 log ${}_{10}$ CFU/mL of viable yeasts were found in the bioreactor. Besides, no significant difference was observed between yeast survival kinetics in the large intestinal conditions for the three donors included in this study.

### 3.3. Influence of Probiotic Yeast on Human Colonic Microbiota Composition and Metabolic Activity

**Figure 4:**
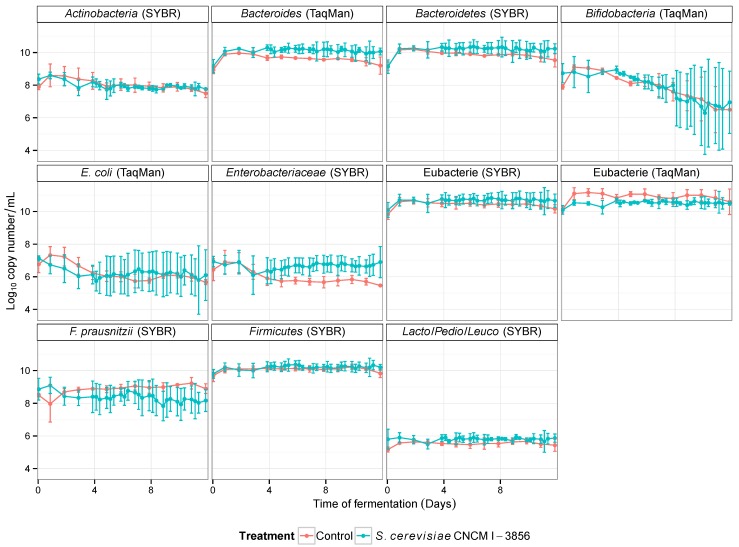
Effect of *S. cerevisiae* CNCM I-3856 on the main populations of colonic microbiota in the ARCOL model. Results are expressed as mean log${}_{10}$ copy number/mL ± the standard deviations (*n* = 3).

**Figure 5:**
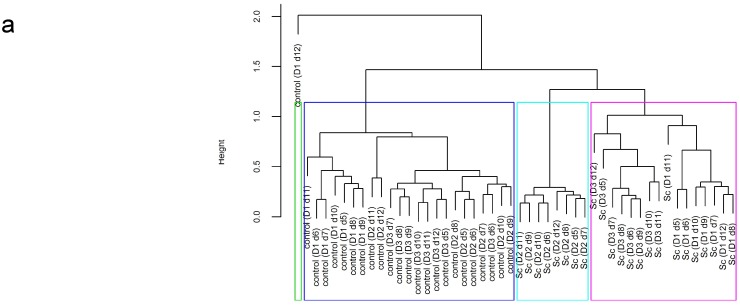

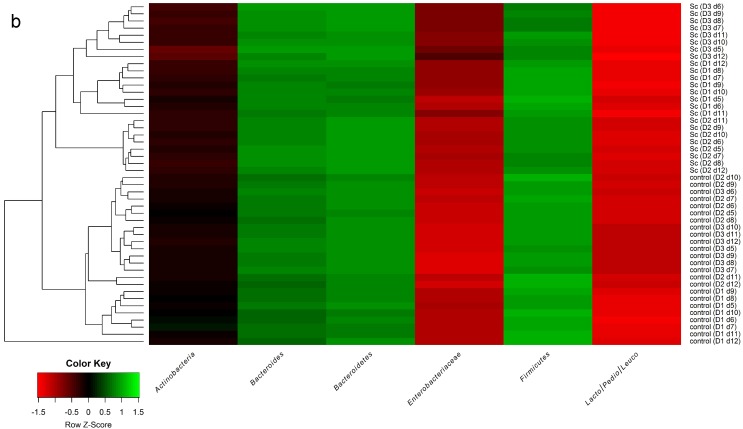
Individual-dependent effect of *S. cerevisiae* CNCM I-3856 on the colonic microbiota in the ARCOL model. An agglomerative hierarchical clustering (**a**) and the resulting heat map (**b**) were made based on the results obtained from the qPCR analysis of the main bacterial groups of colonic microbiota. The labels of the dendrogram indicate for each sample the treatment-donor-day of fermentation. Heat map coloring refers to the concentration values: high values are represented in green, while low values are represented in red.

The major phyla of gut microbiota and their main members were quantified in the ARCOL model by qPCR using 16S rRNA targeted oligonucleotide specific primers. Whatever the time of fermentation and the treatment (control or probiotic treatment with *S. cerevisiae* CNCM I-3856), no significant change was observed in the levels of the selected populations when the data obtained for the three donors were averaged (Figure 4). To further investigate the effect of probiotic supplementation on each individual’s gut microbiota, an agglomerative hierarchical clustering on the major phyla and genus (*Bacteroidetes*, *Firmicutes*, *Actinobacteria*, *Bacteroides*, *Lactococcus*/*Pediococcus*/*Leuconostoc*, *Enterobacteriaceae*) was used to gather the data (Figure 5a). The resulting heat map of clustering shows the individual variations in response to the different treatments (Figure 5b). Irrespective of the stool donors, all control samples (no supplementation) were found in the same cluster. Conversely, *S. cerevisiae* CNCM I-3856 had individual-dependent effects on colonic microbiota. As an example, in Donor 3, yeast treatment led to a decrease in *Lactococcus*/*Pediococcus*/*Leuconostoc* and an increase in *Enterobacteriaceae*. In Donor 2, probiotic treatment led to a decrease in *Bacteroidetes*. Interestingly, Donor 1 and Donor 3, who are male, showed profiles more closely related than that of Donor 2, who is a female. To further investigate the effects of the probiotic on the colonic microbiota, its metabolic activity was followed daily by assessing the production of major and minor SCFAs in the dialysis outflows of the ARCOL model. Whatever the treatment, acetate was the main metabolite produced, then followed by propionate and butyrate (Table 4). The percentages of acetate, propionate and butyrate in the dialysis outflows were around 67%, 19% and 14% of major SCFA, respectively. The yeast treatment did not induce any modification in SCFA production compared to the control experimentations (*p* > 0.05).

**Table 4:** Influence of probiotic treatment on short chain fatty acid production.

SCFA	Control	*S. cerevisiae* CNCM I-3856
Acetate	67.2 ± 3.6	66.9 ± 3.0
Propionate	18.5 ± 2.6	18.7 ± 2.2
Butyrate	14.4 ± 2.0	14.4 ± 1.9
*iso*-Butyrate	1.9 ± 0.3	2.1 ± 0.2
Valerate	4.1 ± 1.0	4.8 ± 0.7
*iso*-Valerate	2.7 ± 0.4	3.0 ± 0.3
Hexanoic acid	2.5 ± 1.2	2.8 ± 1.7
Heptanoic acid	0.3 ± 0.3	0.6 ± 0.6

Data are the mean percentages of total SCFA (defined as the sum of acetate, propionate and butyrate) ± the standard deviations (*n* = 3), for the overall fermentation period.

## 4. Discussion

Survival in the human gastrointestinal tract is generally considered as a key feature for probiotics to preserve their health-promoting effects. However, due to the cost and complexity of *in vivo* studies in human subjects, most of the available data focus on the fecal recovery of probiotics and little is known about their behavior throughout the digestive tract, which is considered as a black box. For ethical, technical, regulatory and financial reasons, *in vitro* digestive models can be used as an alternative to human studies, provided that their relevance has been fully demonstrated compared to the *in vivo* situation [29,30]. In this context, the aim of the present study was to use relevant *in vitro* models of the upper (TIM-1) and lower (ARCOL) gastrointestinal tract to assess the survival in the human digestive environment of the new probiotic yeast *S. cerevisiae* CNCM I-3856. Previous *in vitro* and *in vivo* studies have already established the beneficial effects of this strain [7,8,9], but up to date little is known about its behavior during digestion in human. In particular, there is no published data reporting its survival in humans volunteers.

The probiotic survival rate in the human digestive tract will depend on the means of administration. In particular, probiotic viability is conditioned by the food matrix in which the probiotics are ingested [31,32] and the galenic form for their oral administration [33,34,35]. Since a previous study has already established the effect of dosage forms (capsule or tablet) on the survival rate of *S. cerevisiae* CNCM I-3856 in the TIM-1 model [13], we focused in this work on the effect of food matrix when the probiotic was administered in its active dried powder form. *S. cerevisiae* CNCM I-3856 was introduced into the TIM-1 system within a glass of water or a Western-type meal at a physiological dose [8], and the model was set-up according to *in vivo* data to mimic the fasted and fed states in healthy human adults. The main digestive parameters influenced by food intake, such as drop in gastric pH, half-time of gastric emptying, time of gastrointestinal transit and luminal concentrations of digestive secretions were taken into account in the TIM-1 model [36,37,38,39,40,41]. The probiotic yeast showed a high resistance during its transit through the *in vitro* upper gastrointestinal tract, proving its ability to face stressful environmental conditions such as gastric acidity or bile secretion in the intestine. According to our results, as much as 6.4 log${}_{10}$ ± 0.05 CFU/mL of culturable yeasts are likely to reach the human colon, where they are mostly supposed to exert their health effect on the host [7,8]. The beneficial effects of *S. boulardii* are dependent on the viable yeast concentration in the digestive tract [42]. Even if the minimal dose required for a probiotic effect is still debated, it was suggested that the concentration of cells needed to obtain a clinical effect in the small bowel was quoted to be 10${}^{6}$ CFU/mL[43]. This implies that the concentrations of *S. cerevisiae* CNCM I-3856 reaching the large intestine would be sufficient to exert their potential beneficial effect. In addition, yeast survival in the ileal effluents was not dependent on the fed or fasted conditions. To the best of our knowledge, no studies have investigated the influence of food vehicle on the survival of probiotic yeast in the upper human gastro-intestinal tract. Our results suggest that the probiotic should be indifferently administered under fed or fasted conditions to ensure a high viability when entering the colon. Nevertheless, from 180 min of digestion, probiotic cells were present longer in the jejunum and ileum under the fed state, due to slower transit time. This may have an impact on probiotic activity if the strain has a targeted action in the distal parts of the small intestine.

Once the gastric and small intestinal barriers are crossed, the probiotics have to succeed in competing with the resident colonic microbiota. In this study, a rapid elimination of *S. cerevisiae* CNCM I-3856 from the ARCOL model was noticed, despite a twice daily supplementation with the probiotic. These results suggest that the yeast was strongly affected by the colonic conditions. This extensive elimination may result from the barrier effect of the endogenous microbiota and is fully in line with the available data in humans where *S. boulardii* or other strains of *S. cerevisiae* were eliminated from fecal samples within two to three days after cessation of treatment [44,45]. Our results suggest that the major barrier to the survival of *S. cerevisiae* CNCM I-3856 is not the acidic gastric environment, as previously suggested for *S. boulardii* [46,47], but rather the conditions found in the large intestine.

Since our results indicated that gut microbiota may have a key role in the colonization of *S. cerevisiae* CNCM I-3856, we investigated for the first time the effect of its supplementation on gut microbiota composition and activity. When the results obtained with the three volunteers (one female and two males ranging from 24 to 46 years old) were averaged, we found that the probiotic yeast influenced neither the main populations of the gut microbiota nor the production of SCFAs. Our results are in agreement with those previously published on *S. boulardii* reporting that this yeast has no major effect on the fecal microbiota composition and metabolic activity in healthy subjects [48,49]. Nevertheless, when the effect of *S. cerevisiae* CNCM I-3856 was assessed on each individual microbiota, the response to the probiotic treatment was found to be subject-dependent. Even if the number of donor remains relatively low, this is the first study reporting an individual-dependent effect of a treatment with a probiotic yeast on the human gut microbiota. Therefore, despite a low survival rate under colonic conditions, *S. cerevisiae* CNCM I-3856 seemed to have the capacity to influence the gut microbiota at the individual level. A possible difference between males and females was suggested but has to be confirmed using a larger number of donors. Bolnich *et al.* have shown that microbiota could be influenced by several parameters as diet environment and genotype, sex being just one of many possible genetic polymorphisms [50]. Nevertheless, such a difference in microbial profiles was not linked with any variation in SCFA production between the three volunteers. All of these experimentations have been carried out with feces from healthy subjects. Some studies have shown that *S. boulardii* is able to modulate gut microbial composition under unhealthy conditions, such as in patients with long-term total enteral nutrition [51] or in obese and type 2 diabetic mice [52]. Therefore, it would be of great interest to assess the effect of *S. cerevisiae* CNCM I-3856 in the ARCOL model inoculated with dysbiosis microbiota from diseased individuals (*i.e.*, IBS patients).

## 5. Conclusions

In conclusion, this study shows that dynamic *in vitro* models of the upper and lower gastrointestinal tract such as TIM-1 and ARCOL can provide significant insight into the behavior of probiotic strains during digestion in humans. In particular, we showed that the major barrier in the colonization of the new probiotic strain *S. cerevisiae* CNCM I-3856 was not the acidic environment of the stomach but rather the competition with resident colonic microbiota. The survival of the yeast in the ileal effluents was not influenced by fed or fasted conditions, giving valuable information on the probiotic mode of administration in human subjects. Interestingly, the effect of *S. cerevisiae* CNCM I-3856 on the gut microbiota was shown to be individual-dependent, suggesting that human individuals should respond differentially to the probiotic treatment.
